# Continuous Exposure to Low Doses of Ultrafine Black Carbon Reduces the Vitality of Immortalized Lung-Derived Cells and Activates Senescence

**DOI:** 10.1155/2020/5702024

**Published:** 2020-12-07

**Authors:** M. Esther Salinas, Denisse A. Gutiérrez, Armando Varela-Ramírez, Kristine M. Garza

**Affiliations:** Department of Biological Sciences, Border Biomedical Research Center, University of Texas at El Paso, 500 West University Avenue, El Paso, TX 79968-0519, USA

## Abstract

Combustion-derived nanomaterials are noxious ultrafine (<100 nm) aerosol by-products of human activity. They pose threats to pulmonary health due to their small size, allowing them to penetrate alveoli causing detrimental responses downstream. Information regarding the cellular activity that connects nanocarbon particle exposure to poor pulmonary health remains lacking. We hypothesized that low-dose and long-term administrations of carbonaceous nanoparticles contribute to lung irritation by adversely affecting respiratory cells that function as the first line of defense. Responses to *ultrafine black carbon* (UBC), a key component of airborne pollutants, by human lung A549, murine lung LA4 epithelial cells, human peripheral-blood monocytes THP1, and murine macrophages RAW264.7 were investigated. The cells were first plated on day zero and were fed fresh UBC suspended in culture media on days one, four, and seven. The exposure regimen included three different concentrations of UBC. On day ten, all cells were harvested, washed, and assayed. The impact on cellular viability revealed that UBC was only moderately cytotoxic, while metabolic activity was significantly diminished in a dose-dependent manner. Additionally, beta-galactosidase proportionally increased with UBC concentration compared to untreated cells, indicating that cellular senescence was promoted across all cell types. The implemented regimen caused minimal toxicity yet demonstrated different cellular modifications across the cell lines of both species, inducing changes to enzyme vitality and cellular fitness. The data suggested that compounding nanosized black carbon exposure could negatively impair overall pulmonary health by distinctively modifying intracellular behavior.

## 1. Introduction

Particulate matter (PM) is one of six criteria pollutants; it is a complex mixture of extremely small particles and liquid droplets. According to the Center for Climate & Energy Solutions, PM can originate from natural sources such as forest fires and wind erosion and from human activities like agricultural practices, wood burning, smokestack releases, transportation emissions, and even discharges from construction sites [1]. The United States Environmental Protection Agency (US-EPA) classifies particle pollutants by category: PM_10_ or coarse particles (2.5 *µ*m–10 *µ*m in diameter) such as dust, pollen, or mold; PM_2.5_ or fine particles (<2.5 *µ*m) such as combustion particles from industrial processes, organic compounds, and metals; and PM_0.1_ ultrafine particles (<100 nm) also known as nanosized PM, not only generated from gasoline and diesel exhaust engine by-products but also found transported via heavy dust storms [2]. Inhaling both fine and ultrafine particles pose severe threats to humans, where the lung is the primary target organ; these mixtures of PM particles may lead to or contribute to a vast number of occupational or particle-influenced lung diseases [3].

Human enterprises have remarkably increased the output and by-products of combustion activities, augmenting the occurrence of nanosized PM in the adjacent atmosphere and posing greater health risks. Indeed, ultrafine particles represent almost 94% of the total particle concentrations found in the border crossing airshed at the Bridge of the Americas (BOTA) between Chihuahua, MX, and Texas, the US, for example [4]. While nanosized particles account for <1% of the general airborne mass of PM, they account for a significant fraction (>90%) of PM abundance [5]. Thus, a given mass of ultrafine particles will impact a significantly larger surface area of lung tissue than will an equal mass of bigger particles, intensifying interactions between living organisms and ultrafine aerosol.

Interestingly, the unique physical and chemical properties of nanosized materials alter their interaction with biological tissues licensing them for uptake at the cellular level (macropinocytosis), an uncommon phenomenon for larger materials [6]. Oberdörster et al. indicated that respirable particle deposition is due to diffusion (displacement upon collision in the central airway) for nanosized particles. They estimate that out of the total amount of inhaled particles, the deposition of nanoparticles occurring at the alveolar region is at 50% efficiency [7]. The estimation was further supported with *in vivo* experimental data where the majority of nanoparticles (10–50 nm) inhaled by rats appeared to be retained at this deep gas exchange site. Approximately 80% of the particles were localized at alveoli respiratory membranes, implying that alveolar macrophages cannot clear ultrafine particles even after a 24-hour period [7]. Thus, the interaction between lung tissue and inhaled nanoparticulates is extensive. Further and enhanced delineation of respiratory cellular responses to nanoparticles will provide a greater understanding of particle-associated pulmonary ailments.

The literature on the effects of inhalable aerosols is abundant and suggests a variety of possible mechanisms of how these pollutants cause deleterious human health outcomes [8–16]. However, these studies have shown the harmful effects of short-term (24–48 hours), high dose (10–500 *µ*g/mL) exposure to nanosized carbon, which is an unlikely scenario of human inhalation exposure. Presently, long-term (>1 week), low-dose (<10 *µ*g/mL) cumulative studies exploring the link between chronic pulmonary pathology and ultrafine black carbon (UBC) exposure are lacking. Here, we utilized an *in vitro* exposure model to mirror a closer approximation of cell contact with PM to investigate cellular responses to a cumulative exposure of nanocarbon particles at low concentrations. Cells were exposed to fresh concentrations of UBC (0.3, 1.0, and 3.0 *µ*g/mL) every three days for a total of 9 days. Cellular viability, caspase-3/7 activity, enzyme vitality, and cell senescence after a 9-day period were analyzed to assess the overall impact. Our findings showed that the three low-dose concentrations of UBC administered over a 9-day period were marginally cytotoxic and minimally induced caspase-3/7 activity in an inverse manner. Enzyme function, however, was markedly reduced for all cell lines in an inverse dose-dependent manner. In addition, a state of cellular senescence was induced proportional to UBC doses. Our results indicated that the cells were affected by the compounding treatments of UBC, although exposures were not severely toxic. The 9-day regimen demonstrated that UBC nanoparticles adversely impact mammalian cell function and that each cell line responds uniquely, although all responses ultimately resulted in cellular senescence. These findings further suggested that experimental treatments approximating physiological exposure (low-dose, over time) may differ from acute (high dose) exposures by cell line, hinting that particle effects must be evaluated in a holistic manner to ascertain their underlying mechanisms of action.

## 2. Methods

### 2.1. Carbon Nanomaterials

Black carbon (Vulcan XC-72) purchased from Cabot Corporation (Billerica, MA) was used for our experiments. Carbon nanoparticles, or UBC, have been characterized by Soto et al. by transmission electron microscopy (TEM) using a Hitachi H-8000 analytical TEM operated at 200 kV accelerating potential and fitted with a goniometer-tilt stage, and a Noran Energy Dispersive (X-ray) spectrometer (EDS) [17]. Carbon nanoparticles were suspended at 10 mg/mL in 1x PBS from Fisher Scientific (Pittsburg, PA) and were manipulated to create 1.0 mg/mL stock solutions. These were further diluted in the appropriate cell culture media for the experimental assays and constantly resuspended prior to administration using a vortex (<1 min) to minimize carbon nanoparticle agglomeration.

### 2.2. Cell Lines and Culture Conditions

All four cell lines were obtained from the American Type Culture Collection (ATCC, Manassas, VA). The adherent A549 and LA4 epithelial cells were cultured in F-12K Media (Kahn's), supplemented with 10% fetal bovine serum (FBS) and 1% penicillin-streptomycin from Fisher Scientific (Pittsburg, PA). Adherent murine macrophages RAW264.7 were grown in Dulbecco's Modified Eagle Media (DMEM), supplemented with 10% FBS, 1% penicillin-streptomycin, 1% sodium pyruvate, 1% sodium bicarbonate, and 1% GlutaMAX from Fisher Scientific (Pittsburg, PA). Nonadherent THP1 monocytes were cultured in Roswell Park Memorial Institute (RPMI 1640) media, supplemented with 0.05% *β*-mercaptoethanol, 10% FBS, 1% penicillin-streptomycin, 1% sodium pyruvate, and 1% GlutaMAX from Fisher Scientific (Pittsburg, PA). The incubation conditions were kept at 37°C in a humidified 5% CO_2_ atmosphere across all cell lines.

### 2.3. Exposure Regimen

Assays were conducted with the following five treatment groups: (1) untreated cells; (2) H_2_O_2_-treated cells; (3) 0.3 *µ*g/mL UBC-treated cells; (4) 1.0 *µ*g/mL UBC-treated cells; (5) 3.0 *µ*g/mL UBC-treated cells. On day 0, cells were seeded in 6-well plates (Fisher Scientific, Pittsburg, PA) at a density of 1,500 cells per well and allowed to equilibrate for 24 hours. On day 1, the cells were treated with the three concentrations of UBC; two groups per cell type remained untreated to serve as controls. On both day 4 and day 7, supernatants were removed from wells and fresh UBC-media suspensions (0.3, 1.0, or 3.0 *µ*g/mL UBC in fresh culture media) were readministered to the experimental groups; untreated control groups received just fresh media. On day 9, one set of untreated cells were exposed to hydrogen peroxide (10 mM H_2_O_2_) to serve as a control for toxicity. On day 10, all five groups were harvested, centrifuged, and resuspended in 1x cold PBS and prepared for subsequent assays.

### 2.4. Membrane Integrity Assay

Cell viability was measured by propidium iodide (PI: Fisher Scientific, Pittsburg, PA) dye exclusion assay after 9 days of exposure to nanocarbon particles. This method determined the number of viable cells in suspension as a function of plasma membrane health because PI only stains the nuclei of cells with a compromised membrane. Following 9 days of treatment (see exposure regimen from above), the cells were harvested and transferred to flow cytometry tubes (Fisher Scientific, Pittsburg, PA); each experimental point and their corresponding controls were performed in triplicate. Subsequently, cells were washed, resuspended, and stained with PI at a final concentration of 5.0 *µ*g/mL. The samples were quickly and gently vortexed then read *via* flow cytometry (Beckman Coulter FC500 Flow Cytometer Cytomics Analyzer System). Viability (% of live cells) was determined as the number of PI-negative cells; inversely, PI-positive cells were indicated by % of dead cells.

### 2.5. Apoptosis/Necrosis Assay

The distribution of apoptotic/necrotic cellular profiles was conducted using FITC-Annexin V/PI assay (Beckman Coulter, Miami, FL) and flow cytometry. Following 10 days of UBC treatment (see exposure regimen from above), the cells were harvested and transferred to ice-cold flow cytometry tubes (Fisher Scientific, Pittsburg, PA). The cells were washed with ice-cold PBS and stained with 1.0 *µ*L of FITC-conjugated Annexin V and 5.0 *µ*L of PI diluted in binding buffer (100 *µ*L total volume). Next, cells were incubated on ice in the dark for 15 min, and 400 *µ*L of the ice-cold binding buffer was added. Thereafter, the cells were gently vortexed and analyzed by flow cytometry (Beckman Coulter Cytomics FC500 Flow Cytometer Analyzer System). Each experimental set of tubes along with the associated control tubes were examined concurrently. Approximately 10,000 events (cells) were collected per sample and examined *via* CXP software (Beckman Coulter).

### 2.6. Active Caspase-3/7 Assay

Activation of caspase-3/7 as a measure of apoptosis by flow cytometry protocol after exposure to nanocarbon was evaluated with the NucView® 488 Caspase-3/7 (Biotium, Inc.) assay kit. A cell membrane-permeable fluorogenic caspase reagent was used to detect caspase-3/7 activity within live cells. Following UBC treatment (see exposure regimen), these were collected and homogenized in 500 *µ*L of 1x DMEM phenol-red free media (Gibco). 100 *µ*L of each sample was transferred to flow cytometry tubes (Fisher Scientific, Pittsburg, PA) in replicates and 2.5 *µ*L of caspase reagent was added and dark incubated for 45 min. A 300 *µ*L aliquot of 1x PBS was added to achieve a final caspase reagent concentration of 5 *µ*M. The samples were gently mixed and immediately read with a Gallios Flow Cytometer (Beckman Coulter). Cells emitting a green fluorescent signal denote the activation of caspase-3/7 and are potential apoptotic positive samples. These were analyzed and statistics were conducted against the untreated controls across all cell types. At least three separate experiments in the duplicate form are depicted.

### 2.7. Vitality and/or Enzyme Assays

Biochemical enzyme activity was measured via Cell Titer-Glo, Calcein AM, and *β*-Galactosidase (*β*–Gal) assays after nine days of continuous exposure to nanocarbon. The cells were exposed to UBC, as previously described. Following UBC treatments, the cells were harvested, centrifuged, and resuspended in 1,000 *µ*L of 1x PBS; an aliquot of 50 *µ*L for each sample was transferred to 96-well plates (Fisher Scientific, Pittsburg, PA) in replicates of six. Subsequently, reagents were added according to each different biochemical kit/experiment, listed as follows (parentheses annotate instrument used for each individual assay): Cell Titer-Glo luminescence assay from Promega (Luminoskan Ascent Software v2.6, Thermo Fisher Scientific Ascent Luminometer v2.5); Calcein AM fluorescence assay from BD Pharmingen (Fluoroskan Ascent Software v2.6, Thermo Fisher Scientific Ascent Fluorometer v2.5); Mammalian *β*-Galactosidase (*β*–Gal) colorimetric assay from Thermo Fisher Scientific (SoftMax Pro 5.4.1, Molecular Devices SPECTRAmax 190 Microplate Spectrophotometer). Data for ATPase and serine esterase displayed normalized values relative to the media controls, shown as the mean of six replicates ± standard error bars. Data for senescence displayed normalized values relative to the media controls (100%), shown as the mean of six replicates ± max and min values.

### 2.8. Statistical Analysis

Significant differences between both control and experimental groups were evaluated using R Project for Statistical Computing by applying a one-way ANOVA with a post hoc Tukey test at a significance level of *∗p* < 0.05. If the data was not normally distributed, a nonparametric Kruskal–Wallis with a nonparametric post hoc multiple comparisons test (significance level *∗p* < 0.05) was conducted to determine statistical significance. GraphPad Prism (Project Software v5, San Diego, CA) was used to create graphical representations of the average values for the data collected (with ±SE bars for enzyme vitality assays and ±max and min bars for senescence assay).

## 3. Results

To evaluate the potential impact of airborne PM on the lungs at the cellular level, an *in vitro* approach using human and murine epithelial and immune cells was optimized. The cell lines tested include human pulmonary epithelial cells (A549), human peripheral-blood monocytes (THP1-macrophage precursors), murine macrophages (RAW264.7), and murine pulmonary epithelial cell (LA4) lines. These lines were chosen because epithelial cells and macrophages are the first to come in contact with inhaled particulate matter, and both can contribute to stress and inflammation of the pulmonary tissues [18]. Several studies have shown that airway epithelial cells express on their surface adhesion molecules and secrete various immune molecules such as chemokines [19–22]. Of importance, macrophages are the most abundant immune cells in the lung and play an essential role in the systemic immunity of the lung [23]. Excessive or unwanted activation of macrophages has been implicated in respiratory disorders like chronic obstructive pulmonary disease (COPD) and other persistent ailments such as bronchitis and asthma [24]. Our aim was to evaluate the responses of lung-derived cells to accumulating levels of UBC. The *in vitro* model allowed for controlled administration of the carbon nanomaterial at lower than typically published concentrations and for longer than typically published exposure times [25].

### 3.1. Morphology

Ultrafine black carbon (UBC) was utilized as a model for environmental particle insult. As published in 2006, the diameter “for any fuel combustion-producing” particulate consists of nanostructures whose primary carbonaceous spherule ranges from 15 to 70 nm in size [26]. Soto et al. confirmed that black carbon aggregates found in wood and diesel soot are identical in their structures to commercially prepared carbon nanoaggregates [17]. The UBC utilized for these studies was the exact same source as that described in [26]; thus, the diameter range of UBC aggregates utilized for our studies was approximately 15–70 nm. Moreover, as indicated by the manufacturer, the nanomaterial was endotoxin-free.

**Figure 1:**
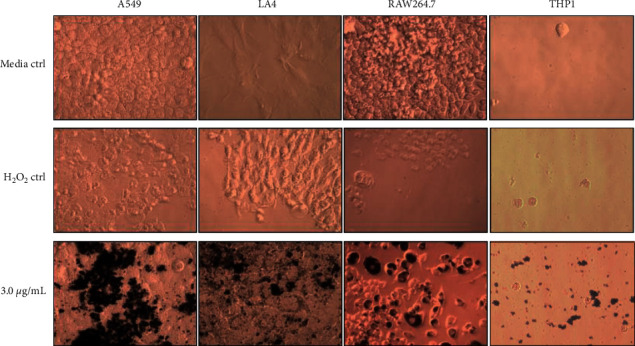
Low-dose UBC aggregates on the cell surface and may translocate into macrophages. Images captured upon UBC treatments after the 9-day mark across A549, LA4, RAW264.7, and THP1 cell lines. The images were digitally captured by light microscopy using a Leica Model DMIL EC3, LED and observed with LAS EZ Leica Microsystems program at a 40x magnification: media “untreated” control, 10 mM H_2_O_2_ control, and 3.0 *µ*g/mL UBC are shown across each cell type.

The immediate impact of UBC exposure on the cells was recorded by capturing light microscopy images posttreatment to visualize cellular morphology and UBC localization (Figure 1). The cells were plated on day 0; on day 1, UBC in fresh media was administered. On days 4 and 7, media and suspended UBC were removed from the cells and fresh UBC-media suspensions were provided. On day 9, the cells were photographed. Overall, the UBC-exposed cells were relatively healthy. The morphology of both human and murine epithelial cells was largely unaffected; however, UBC appeared to preferentially aggregate on the surface of these cells forming carbon particle clusters. Aggregation was particularly dense for the human epithelial cells. In comparison to earlier time points (data not shown), the density of aggregates appeared to increase with each treatment. As expected, murine macrophages appeared to internalize the higher amounts of UBC and were also enlarged. Human monocytes exhibited internalization of UBC at much lower densities. As with the epithelial cells, the density of UBC increased with time, particularly for the murine macrophages (data not shown). Although the photomicrographs depict curious UBC-cell interactions, the scope of this paper neither presumes to answer nanoparticle measurements nor nanostructure dispersion upon cellular interactions. More work is certainly needed to describe this behavior in a noninvasive method that does not compromise the integrity of any cells or tissues in question [27].

### 3.2. Viability

**Figure 2:**
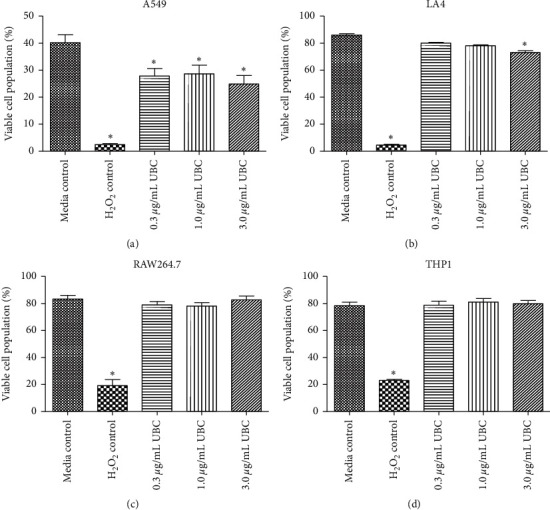
UBC minimally impacts viability as measured by cellular membrane integrity. Cells were treated as described in Methods. These were harvested and cell viability was monitored *via* flow cytometry as a function of PI after day 9. The number of live cells (viable) is shown as the percentage of PI-negative cells for each cell line ((a), (b), (c), and (d)). Data determined as the mean of six replicates and is representative of three independent experiments. Statistically significant at *∗p* < 0.05 in comparison to media controls (untreated).

To assess the cytotoxic impact of long-term and low-dose exposure to UBC, plasma membrane integrity was evaluated *via* the fluorescent vital exclusion dye propidium iodide (PI). PI, a DNA intercalating reagent, is a highly charged molecule that does not travel across the cell membrane of living cells [28]. Autofluorescent PI binding to DNA serves as an indicator of dead or dying cells. As shown in Table 1 and Figure 2, the highest concentration of UBC exposure (3.0 *μ*g/mL) presented less viable cells in comparison to the media control for epithelial cells (Figures 2(a) and 2(b)). Little to no change in the percentage of viable cells was observed in comparison to matched untreated controls for the macrophages and monocytes (Figures 2(c) and 2(d)). Thus, using a PI fluorescent marker for membrane integrity as a measure of toxicity, the UBC-treated cells were, for the most part, healthy.

**Figure 3:**
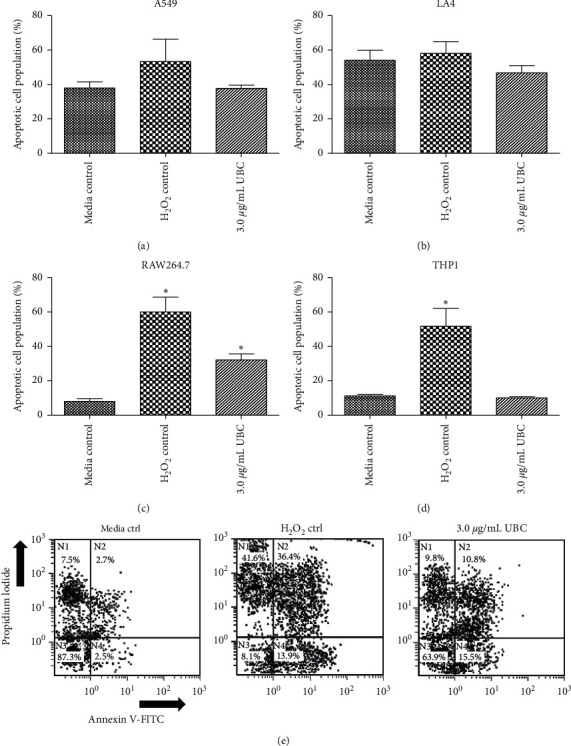
UBC induces low to moderate apoptotic conditions, as shown by FITC-Annexin V/PI costaining. All cells were seeded on day zero then treated with three concentrations of UBC, as described in Methods. Following day 9, the cells were harvested and apoptosis/necrosis was assessed by flow cytometry after double staining with FITC-Annexin V and PI. Percentage of apoptotic cells are shown for each of the cell lines ((a), (b), (c), and (d)) upon treatment with the highest UBC concentration. A representative double-parameter flow cytometry dot-plot for RAW 264.7 is presented (e) where *N1* shows necrotic cells that are PI-positive, but FITC-Annexin V negative; *N2* depicts late apoptotic cells that are FITC-Annexin V and PI double-positive; *N3* specifies unstained viable cells with intact membranes that are FITC-Annexin V and PI double-negative; *N4* contains early apoptotic cells that are PI-negative yet FITC-Annexin V positive. Dots (or events) show density quadrants. Low-density regions are designated in a lighter number of events, high-density regions in the darker number of events. Approximately 10,000 events were acquired per sample. Data determined as the mean of six replicates and is representative of three independent experiments. Statistically significant at *∗p* < 0.05 in comparison to media (untreated) controls.

FITC-Annexin V/PI costaining experiments were conducted to further evaluate the possibility of UBC-induced cell death via an apoptotic or necrotic mechanism. Annexin V specifically binds to phosphatidylserine (PS), which is favorably localized in the inner membrane leaflet of a live/healthy cell. In apoptotic cells, PS is translocated to the outer membrane leaflet allowing for the discrimination between apoptotic and necrotic cells with the utilization of fluorescein isothiocyanate (FITC) conjugated Annexin V. Cell membrane integrity and viability profiles were evaluated by observing cell distributions as follows: early apoptosis, intact membrane with phosphatidylserine externalized positive to FITC-Annexin V; late apoptosis, permeable membrane and positive to both FITC-Annexin V and PI; necrosis, loss of membrane integrity, positive to PI, but negative to FITC-Annexin V. In reference to the dot-plots shown in Figure 3(e), each quadrant indicates cell conditions: left top *(N1)* denotes necrotic cells, right top *(N2)* depicts late apoptotic cells, left bottom *(N3)* specifies viable cells with intact membranes, and right bottom *(N4)* shows early apoptotic cells. The percentages of apoptotic cells upon nine-day UBC treatment was not significantly different for A549, LA4, or THP1 cells across all UBC concentrations (3.0 *μ*g/mL UBC data is displayed), as shown in Figures 3(a), 3(b), and 3(d); apoptotic profiles statistically increased only at the 3.0 *μ*g/mL UBC-treated RAW264.7 macrophages (Figure 3(c)). See Table 1 for supplementary information. As a measurement of cell viability, the apoptosis/necrosis assay showed that only the murine RAW264.7 were significantly sensitive to UBC-induced cytotoxicity.

### 3.3. Caspase-3/7 Activation

**Figure 4:**
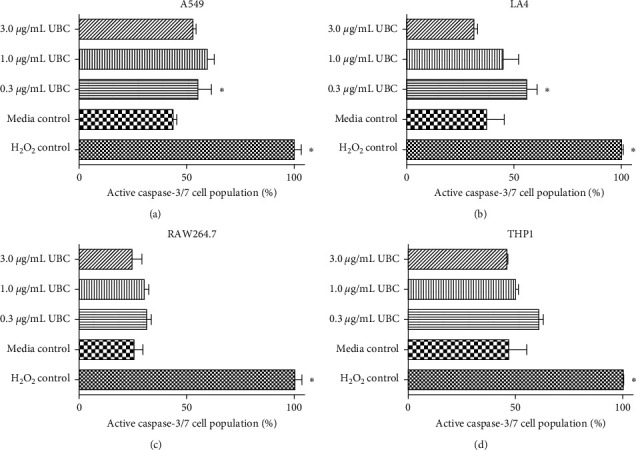
UBC induces little to no apoptosis as measured by caspase-3/7 activity. Cells were UBC-treated as described in Materials and Methods. On day 9, the cells were harvested and stained for active caspase-3/7 for flow cytometric analysis. The data were normalized to the positive control for apoptotic death (hydrogen peroxide-treated cells) for each individual cell line, ((a), (b), (c), and (d)). Data are shown as the mean of two-three duplicates and are representative of three independent experiments. Statistically significant at *∗p* < 0.05 in comparison to the untreated controls.

To further evaluate the possibility of low-dose UBC induction of cytotoxicity, the activation of caspase-3/7 was assessed. Caspase-3/7 is activated in programmed cell death (or apoptosis) both by extrinsic (death receptor ligand) and intrinsic (mitochondrial) pathways, thus serving as a convergence point for the two different mechanisms. As the executioner caspase, caspase-3 has virtually no activity until it is cleaved by an initiator caspase after apoptotic signaling events have occurred [29]. Activation of caspase-3 can function as a key indicator of apoptotic death. On day 9 of exposure, the UBC-treated cells were harvested and stained for the presence of active caspase-3/7. As shown in Figure 4, for the human and murine epithelial cells (A549 and LA4), the lowest concentration of UBC (0.3 *μ*g/mL) induced a statistically significant increase in apoptosis as a function of caspase-3/7 activation relative to the untreated control (Figures 4(a) and 4(b)). However, 1.0 and 3.0 *μ*g/mL of UBC did not. Moreover, none of the UBC concentrations promoted apoptosis in human monocytes or murine macrophages, THP1 and RAW264.7 cell lines, respectively (Figures 4(c) and 4(d)). Although the change in active caspase-3/7 was not statistically relevant in all cell types, a minor inverse relationship between UBC concentration and active caspase-3/7 content was observed across all species. Nonetheless, the caspase-3/7 data aligned with the viability data in that the low-level UBC treatments did not appear to be noticeably promoting an apoptotic death in any of the cells evaluated in this study.

### 3.4. Enzyme Vitality

**Figure 5:**
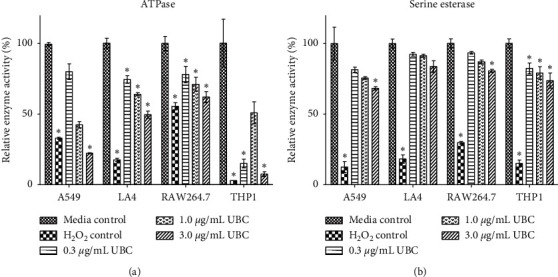
UBC compromises mitochondrial ATPase and cytoplasmic serine esterase activity in a dose-dependent manner across all four cell types. Cells were seeded on day zero then treated with various concentrations of UBC. The cells were harvested and assessed for vitality after day 9 based on (a) mitochondrial ATPase activity (Cell Titer-Glo, Luminescence Assay) and (b) cytoplasmic serine esterase activity (Calcein AM, Fluorescence Assay). Data normalized to the untreated media control and displayed as the mean of six replicate wells ±SE. Normalized activities are indicated as one of five representative independent experiments. Statistically significant at *∗p* < 0.05 in comparison to media (untreated) controls.

Overall cellular viability was moderately altered by the UBC exposure regimen, demonstrating that at these low carbonaceous particle doses, UBC is not overtly cytotoxic. However, a closer look into intracellular behavior was needed to decipher additional parameters that could be susceptible to UBC effects. Therefore, the biochemical activity of two cellular enzymes (metabolic vitality) was investigated upon exposure to long-term, low-dose treatments of UBC at various concentrations: mitochondrial ATPase and cytoplasmic serine esterase seen as in Figures 5(a) and 5(b), respectively. All cell lines treated with 3.0 *μ*g/mL UBC presented a statistically significant decrease in mitochondrial ATPase activity in comparison to the untreated (media) controls, shown in Figure 5(a). The serine esterase assay showed significantly compromised enzyme activity at the 3.0 *μ*g/mL UBC concentration across all cell lines, with the exception of LA4, shown in Figure 5(b). Generally, the four cell lines showed an inverse dose-dependent change in enzyme metabolic activity (or vitality) with increasing concentrations of UBC. No statistical significance was detected for esterase activity in the UBC-treated LA4 murine epithelial cells. Of importance, the enzyme vitality measurements (Figure 5) were in stark contrast to the data for cellular viability (Figures 2 and 3).

### 3.5. Senescence

**Figure 6:**
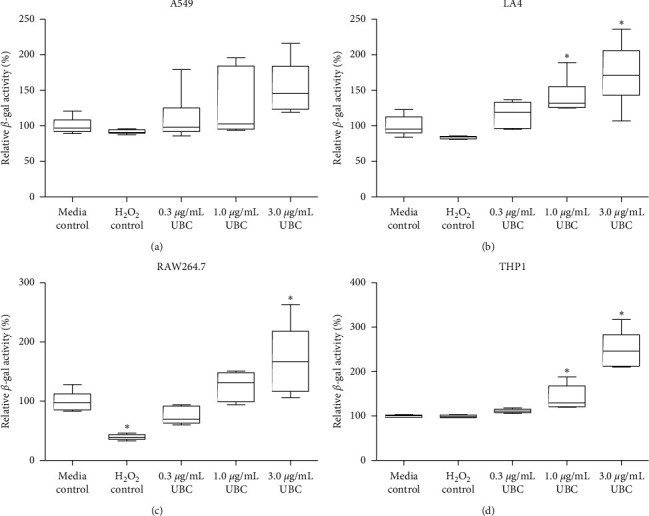
UBC promotes cellular senescence, enhancing beta-Galactosidase (*β*-Gal) activity in a dose-dependent manner for all cell lines. Cells were seeded on day zero then treated with various concentrations of UBC for 9 days. Following subsequent treatments, the cells were harvested and assessed for cellular dormancy following the manufacturer's instructions for the mammalian *β*-Galactosidase kit (*β*-Gal Senescence, Colorimetric Assay). Data normalized to the media control and displayed as the mean of six replicates ±max and min values. Data are indicated as one of three representative independent experiments. Statistically significant at *∗p* < 0.05 in comparison to untreated media controls. (a). A549, (b). LA4, (c). RAW264.7, (d). THP1.

With the cell lines presenting a remarkable phenotype of modest, although varied impact on cellular health, a marked decrease in biochemical activity was identified. Therefore, the possibility of activating cellular senescence was investigated. Cellular senescence is a phenomenon in which cells cease to divide, and although they can no longer proliferate, they remain metabolically active [30]. Senescent cells are uniquely distinct from cells in G_0_ in that an immunogenic and proinflammatory phenotype also stains positive for senescence-associated *β*-galactosidase (*β*-Gal) activity [30]. As presented in Figure 6, the data showed a prominent induction of *β*-Gal upon increasing concentrations of UBC. Specifically, the LA4, RAW264.7, and THP1 cell lines treated with the highest concentration of UBC (3.0 *μ*g/mL) were significantly different compared to the corresponding untreated (media) controls (Figures 6(b)–6(d)). In general, all cell types showed a similar behavior: directly proportional increment of *β*-Gal after nine days of UBC treatments. No statistical significance was detected in the human A549 epithelial cells.

## 4. Discussion

The lack of data supporting the relationship between ambient particle exposure at low and continuous doses with cellular behavior compelled the completion of this 9-day *in vitro* study. The relatively prolonged and cumulative exposure of human (A549) and murine (LA4) epithelial cells, human peripheral-blood monocytes (THP1), and murine macrophages (RAW264.7) to UBC was investigated. Light microscopy revealed viable cells with an overlay of the pollutant on the surface of the cells, yet without any noticeably toxic characteristics. Interestingly, the aggregation of nanomaterial was more intense for human epithelial cells versus murine epithelial cells and appeared to be internalized by the macrophages and somewhat by the monocytes indicating unique cell-UBC interactions for each cell line. Despite the minimal cytotoxic effects of UBC exposure, we surmised nonetheless that UBC would deleteriously affect lung-derived cell lines due to ultrafine black carbon's extremely small size.

Studies were first conducted to determine an optimal incubation time point for untreated cells to remain healthy. Three different intervals (6, 9, and 13 days) and six concentrations (1.0, 3.0, 6.0, 12.5, 25, and 50 *µ*g/mL) of UBC were chosen for our initial assessment. Based on the results, the 9-day interval and UBC concentrations of 0.3, 1.0, and 3.0 *µ*g/mL (from a sonicated stock solution of 1.0 mg/mL in 1X PBS) suspended in cell culture media were selected for the completion of all future experiments. The 9-day time point was selected so as to fall well beyond the stereotypical 24–48-hour exposure conducted to evaluate cytotoxicity. Of note, results for the 9-day and 13-day intervals were similar indicating that for our assessments, 9 days of exposure maximized potential UBC impact. The three UBC concentrations were selected to evaluate dose-response and were based on previously published data where our lower concentrations more appropriately mimicked potential pollutant presence in the lung [31]. It is noted, however, that with three treatments, two of which were replenishments, the working concentrations of the UBC increased over the course of the exposure regime. Nonetheless, even the cumulative dose (0.9 to 9.0 *μ*g/mL) was at least one log lower than other *in vitro* models suggesting that our findings uniquely contribute to understanding nanopollutants' impact on respiratory immune cells.

Measurement of cellular viability after low-dose exposure to UBC at the nine-day mark presented unexpected outcomes. Cellular viability via PI staining showed that low levels of UBC treatments were only moderately cytotoxic or subcytotoxic. As reflected by flow cytometry, membrane integrity assays revealed similar results between the UBC-treated and untreated media controls. No statistically significant difference in the percentage of PI-negative cells in comparison to the untreated controls was observed for murine macrophages (Figure 2(c)) and monocytes (Figure 2(d)). Costaining with PI and FITC-Annexin V was followed to find the percentage of cell populations undergoing both stages of early and late apoptosis, which have a compromised inner membrane leaflet indicating PS externalization [32]. The data yielded a statistically significant increase in programmed cellular death, or apoptosis but only for the RAW264.7 murine macrophages. Therefore, based on membrane integrity and induction of apoptosis, most of the cell lines were not killed by UBC exposures over a nine-day period; only the phagocytic cell line RAW264.7 presented with sensitivity. Thus, low-level UBC treatments did not appear to be overtly toxic to most of the exposed cell types.

To further evaluate the nuances of viability, the potential induction of apoptosis by UBC exposure was considered at a molecular level by assessing the cellular activity of caspase-3/7. As shown in Figure 4, the molecular data mirrored that of the cellular data, indicating that the four tested immortalized cell lines are minimally affected by a 9-day continuous low-dose exposure of UBC. Active (or positive) caspase-3/7 levels were statistically higher for both human and murine epithelial cell lines but only at the lowest UBC concentration. This statistical difference, coupled with the slight inverse relationship between active caspase-3/7 and UBC dose, suggested that the cells might be adjusting to the chronic exposure where the higher doses induced smaller deviations in the cells regarding viability. Although the changes in active caspase-3/7 were not statistically relevant for all cell types, the caspase-3/7 data align with the viability data in which the low-level UBC treatments were not susceptible to an apoptotic death. Again, the data indicated that low-level UBC treatments do not promote extreme cytotoxicity for any of the tested cell lines *via* positive caspase-3/7 activation.

The evaluation of other aspects of cellular activity needed to be performed to elucidate any additional damage that UBC caused to the function of UBC-treated. The low-dose administration of nanocarbon was not inducing cell death in an extreme fashion. However, intracellular metabolic assays suggested a marked decrease in enzyme vitality with increasing concentrations of UBC. ATPase (a mitochondrial enzyme) catalyzes the dephosphorylation of ATP and serine esterase (a cytoplasmic proteolytic enzyme) catalyzes the hydrolysis of esters. Both are critical enzymes that promote normal cell behavior and maintain homeostasis, often harnessing energy vital for subsequent reactions. Interestingly, ATPase and serine esterase activity decreased in all four in a dose-dependent manner. Upon treatments with UBC, all cell lines showed a statistically significant decrease in mitochondrial ATPase at the highest UBC concentration in comparison to the untreated controls. ATPase was significantly lower across all concentrations of UBC in both murine LA4 and RAW264.7 phagocytic cells. Cytoplasmic serine esterase activity was affected in three of the four cell lines and was also significantly compromised with the highest UBC exposure at 3.0 *μ*g/mL. In particular, serine esterase activity was lost across all cell lines compared to the untreated control and statistically significant following all three UBC doses only for the human THP1 monocytes. These adverse effects on internal vitality, which were somewhat unique to each cell line, hint at the notion that enzyme functionality must be closely evaluated as a separate variable from viability when assessing the cellular impact of nanoparticle exposure.

The distinct UBC-inflicted impact observed in the enzyme vitality data could subsequently induce dysfunctional intracellular mechanisms; *in vitro* senescence in cultured cells was tested to reveal repercussions to normal metabolic function after prolonged exposure to nanocarbon. A reduction of enzyme activity is often an occurrence associated with cells that are undergoing activation of a cellular senescent stage [33]. Senescence, which is often an indicator for the targeted secretion of proinflammatory proteins potentially leading to chronic inflammation [34], is a cell condition where the permanent cell cycle arrest occurs in response to cellular stresses [35].

An increased level of cellular *β*-galactosidase (*β*-Gal) activity is a hallmark characteristic of senescent cells [26]. Thus, to evaluate the potential induction of a senescence state in UBC-treated cells, senescence-associated mammalian *β*-galactosidase activity was measured. In general, all cell lines exposed to UBC showed a similar behavior: a directly proportional increase in the response of *β*-Gal activity concomitant to the UBC-gradient concentrations tested. LA4, RAW264.7, and THP1 cell lines treated with 3.0 *μ*g/mL UBC were significantly different compared to their corresponding untreated cell controls. As compared to the untreated cells (media control), findings exhibited an elevated *β*-Gal activity of 1.8-fold for A549 epithelial cells, 2-fold for LA4 epithelial cells and RAW264.7 phagocytes, and 2.5-fold for THP1 monocytes at the highest UBC concentration (3.0 *μ*g/mL). All cellular populations showed a similar behavior: a dose-dependent increase of *β*-Gal, a hydrolase enzyme, after a prolonged period of nanocarbon irritant against both adherent and nonadherent cells. In general, the data showed that low-dose UBC particles elicit senescence across all cell lines included in this study due to an increment of *β*-Gal, suggesting that they are not completely or significantly dying but rather seem to be experiencing proliferation arrest associated with selective changes to an intracellular biochemical activity.

The data clearly does not deliver a mechanism by which the 9-day UBC cumulative exposure induces the observed cellular changes. However, the observations are unique and clearly indicate a slight departure from previously published findings. Of particular interest is the lack of cell death and induction of cellular senescence. The experimental outcomes do not, by any means, point out a cause and effect between pollutant exposure and pulmonary disease but the potential association with senescence as the prospective link is quite provocative.

As previously mentioned, cellular senescence is an irreversible state of permanent cell cycle arrest, occurring in response to multiple noxious insults. During senescence, cells communicate with their surrounding microenvironment by secreting various cytokines and growth factors [37]. This is termed senescence-associated secretory phenotype or *SASP* [38]. SASP is responsible for assembling, secreting chemokines, growth factors, proteases, and senescent cells, yet promoting tissue deterioration [39]. Thus, cellular senescence presents two problems. First, senescence causes a loss of tissue-repair capacity because of cell cycle arrest in progenitor cells, and second, senescent cells produce proinflammatory and matrix-degrading molecules. Thus, the potential link between nanocarbon pollutant exposure and pulmonary ailments may be cellular senescence where low-dose exposure to nanoparticulate matter over a long period of time induces cellular senescence in pulmonary cells potentially contributing to associated pulmonary ailments.

## 5. Conclusion

Long-term, cumulative effects were evaluated *in vitro* to investigate different aspects of cellular behavior to find how cells are affected by ambient pollutants, specifically by unaltered (pristine), amorphous black carbon nanoparticles. Relatively low-dose, long-term UBC exposure killed a limited number of cells and promoted a resting state in the remaining distribution profile. The remaining cells were less biochemically active as a whole, in contrast, and appeared to adapt to the stress by entering an arrested state. The data suggests that as we approach more physiological concentrations of ambient pollutants, these are not likely to induce acute cytotoxicity but instead kill a small population of the exposed cells (the extent to vary depending on cell type). Furthermore, the low-dose, long-term exposure may perhaps render the remaining subpopulation vulnerable to additional chronic insults due to the senescent state. Additional insults to the already vulnerable lung cells could possibly cause a series of downstream proinflammatory responses leading to or predispose to pulmonary disease [40].

In conclusion, exposure to low-dose nanomaterials over an extended period of time likely sensitizes cells, potentially predisposing the local cellular microenvironment to secondary inflammatory insults. UBC exposure *in vitro* negatively affects pulmonary cells and due to its diverse alterations across different cell types, the impact on overall pulmonary health could be more complex than anticipated since elicited pathologies may be harder to predict or treat in human subjects. Thus, persistent exposure to polluted air containing suspended nano-PM may trigger pulmonary ailments associated with anthropogenic activity as a result of biochemical cellular changes.

## Figures and Tables

**Table 1:** Percentage of cell populations displaying viable and apoptotic trends conducted via flow cytometry.

		PI (−) viability	FITC-annexin V (+) apoptosis
A549	Media Ctrl	40.17 ± 2.95	37.92 ± 3.60
	H_2_O_2_ Ctrl (10 mM)	2.42 ± 0.40^*∗*^	53.33 ± 12.93
	0.3 *μ*g/mL UBC	27.83 ± 2.76^*∗*^	36.08 ± 1.11
	1.0 *μ*g/mL UBC	28.67 ± 3.13^*∗*^	35.83 ± 1.54
	3.0 *μ*g/mL UBC	24.92 ± 3.12^*∗*^	37.70 ± 1.79
LA4	Media Ctrl	86.00 ± 1.10	54.08 ± 5.84
	H_2_O_2_ Ctrl (10 mM)	4.50 ± 0.67^*∗*^	58.00 ± 6.83
	0.3 *μ*g/mL UBC	80.08 ± 0.60	49.60 ± 4.14
	1.0 *μ*g/mL UBC	78.17 ± 0.73	43.70 ± 3.57
	3.0 *μ*g/mL UBC	73.17 ± 1.22^*∗*^	46.30 ± 4.09
RAW 264.7	Media Ctrl	83.33 ± 2.69	8.00 ± 1.63
	H_2_O_2_ Ctrl (10 mM)	19.17 ± 4.48^*∗*^	60.00 ± 8.69^*∗*^
	0.3 *μ*g/mL UBC	79.13 ± 2.43	19.17 ± 0.79
	1.0 *μ*g/mL UBC	78.13 ± 2.52	20.33 ± 1.41
	3.0 *μ*g/mL UBC	82.67 ± 2.88	32.17 ± 3.48^*∗*^
THP1	Media Ctrl	78.33 ± 2.64	11.17 ± 0.87
	H_2_O_2_ Ctrl (10 mM)	23.00 ± 0.97^*∗*^	51.67 ± 10.42^*∗*^
	0.3 *μ*g/mL UBC	78.83 ± 2.88	10.67 ± 0.84
	1.0 *μ*g/mL UBC	81.17 ± 2.53	8.83 ± 0.75
	3.0 *μ*g/mL UBC	79.83 ± 2.46	10.00 ± 0.68

^*∗*^Statistically significant at ^*∗*^*p* < 0.05 in comparison to media controls (untreated).

## Data Availability

All data (microscopy, flow cytometry, and colorimetric plate assays) used to support the findings of this study are included within the article.

## References

[1] Climate Basics (2018). *Center for Climate and Energy Solutions*.

[2] Murr L. E., Garza K. M. (2009). Natural and anthropogenic environmental nanoparticulates: their microstructural characterization and respiratory health implications. *Atmospheric Environment*.

[3] Upadhyay S., Ganguly K., Stoeger T. (2014). Inhaled ambient particulate matter and lung health burden. *European Medical Journal Respiration*.

[4] Olvera H. A., Lopez M., Guerrero V., Garcia H., Li W.-W. (2013). Ultrafine particle levels at an international port of entry between the US and Mexico: exposure implications for users, workers, and neighbors. *Journal of Exposure Science & Environmental Epidemiology*.

[5] Oberdörster G. (2000). Pulmonary effects of inhaled ultrafine particles. *International Archives of Occupational and Environmental Health*.

[6] Nel A., Xia T., Mädler L., Li N. (2006). Toxic potential of materials at the nanolevel. *Science*.

[7] Oberdörster G., Oberdörster E., Oberdörster J. (2005). Nanotoxicology: an emerging discipline evolving from studies of ultrafine particles. *Environmental Health Perspectives*.

[8] Chaudhuri I., Fruijtier-Pölloth C., Ngiewih Y., Levy L. (2018). Evaluating the evidence on genotoxicity and reproductive toxicity of carbon black: a critical review. *Critical Reviews in Toxicology*.

[9] de Haar C., Hassing I., Bol M., Bleumink R., Pieters R. (2005). Ultrafine carbon black particles cause early airway inflammation and have adjuvant activity in a mouse allergic airway disease model. *Toxicological Sciences*.

[10] Horie M., Kato H., Fujita K., Endoh S., Iwahashi H. (2011). In vitro evaluation of cellular response induced by manufactured nanoparticles. *Chemical Research in Toxicology*.

[11] Jackson P., Hougaard K. S., Boisen A. M. Z. (2012). Pulmonary exposure to carbon black by inhalation or instillation in pregnant mice: effects on liver DNA strand breaks in dams and offspring. *Nanotoxicology*.

[12] Porter D. W., Hubbs A. F., Chen B. T. (2012). Acute pulmonary dose-responses to inhaled multi-walled carbon nanotubes. *Nanotoxicology*.

[13] Valavanidis A., Vlachogianni T., Fiotakis K. (2016). Air pollution as a significant cause of diseases and premature death. ambient air pollution in urban areas and indoor air pollution are associated with adverse health effects and premature mortality. *Health and Safety in the Working Environment*.

[14] Yamawaki H., Iwai N. (2006). Mechanisms underlying nano-sized air-pollution-mediated progression of atherosclerosis. *Circulation Journal*.

[15] Yang M., Flavin K., Kopf I. (2013). Functionalization of carbon nanoparticles modulates inflammatory cell recruitment and NLRP3 inflammasome activation. *Small*.

[16] Zhu Y., Armstrong J. L., Tchkonia T., Kirkland J. L. (2014). Cellular senescence and the senescent secretory phenotype in age-related chronic diseases. *Current Opinion in Clinical Nutrition and Metabolic Care*.

[17] Soto K. F., Carrasco A., Powell T. G., Garza K. M., Murr L. E. (2005). Comparative in vitro cytotoxicity assessment of some manufactured nanoparticulate materials characterized by transmission electron microscopy. *Journal of Nanoparticle Research*.

[18] Espinosa V., Rivera A. (2016). First line of defense: innate cell-mediated control of pulmonary aspergillosis. *Frontiers in Microbiology*.

[19] Beck-Schimmer B., Schimmer R. C., Pasch T. (2004). The airway compartment: chambers of secrets. *Physiology*.

[20] Madjdpour C., Jewell U. R., Kneller S. (2003). Decreased alveolar oxygen induces lung inflammation. *American Journal of Physiology-Lung Cellular and Molecular Physiology*.

[21] Mayer A. K., Muehmer M., Mages J. (2007). Differential recognition of TLR-dependent microbial ligands in human bronchial epithelial cells. *The Journal of Immunology*.

[22] Pichavant M., Taront S., Jeannin P. (2006). Impact of bronchial epithelium on dendritic cell migration and function: modulation by the bacterial motif KpOmpA. *The Journal of Immunology*.

[23] Mitchell L. A., Gao J., Wal R. V., Gigliotti A., Burchiel S. W., McDonald J. D. (2007). Pulmonary and systemic immune response to inhaled multiwalled carbon nanotubes. *Toxicological Sciences*.

[24] Raven P., Johnson G., Mason K. A. (2017). *Biology*.

[25] Bahadar H., Maqbool F., Niaz K., Abdollahi M. (2016). Toxicity of nanoparticles and an overview of current experimental models. *Iranian Biomedical Journal*.

[26] Murr L. E., Guerrero P. A. (2006). Carbon nanotubes in wood soot. *Atmospheric Science Letters*.

[27] Powers K. W., Palazuelos M., Moudgil B. M., Roberts S. M. (2007). Characterization of the size, shape, and state of dispersion of nanoparticles for toxicological studies. *Nanotoxicology*.

[28] Robles-Escajeda E., Lerma D., Nyakeriga A. M. (2013). Searching in mother nature for anti-cancer activity: anti-proliferative and pro-apoptotic effect elicited by green barley on leukemia/lymphoma cells. *PloS One*.

[29] McIIwain D. R., Berger T., Mak T. W. (2013). Caspase functions in cell death and disease. *Cold Spring Harbor Perspectives in Biology*.

[30] Kirkland J. L., Tchkonia T. (2017). Cellular senescence: a translational perspective. *EBioMedicine*.

[31] Oberdörster G., Castranova V., Asgharian B., Sayre P. (2015). Inhalation exposure to carbon nanotubes (CNT) and carbon nanofibers (CNF): methodology and dosimetry. *Journal of Toxicology and Environmental Health, Part B Critical Rev*.

[32] Robles-Escajeda E., Das U., Ortega N. M. (2016). A novel curcumin-like dienone induces apoptosis in triple-negative breast cancer cells. *Cellular Oncology*.

[33] Campisi J. (2003). Cellular senescence and apoptosis: how cellular responses might influence aging phenotypes. *Experimental Gerontology*.

[34] Campisi J., Andersen J. K., Kapahi P., Melov S. (2011). Cellular senescence: a link between cancer and age-related degenerative disease?. *Seminars in Cancer Biology*.

[35] Sharpless N. E., Sherr C. J. (2015). Forging a signature of in vivo senescence. *Nature Reviews Cancer*.

[36] Hooten N. N., Evans M. K. (2017). Techniques to induce and quantify cellular senescence. *Journal of Visualized Experiments: Journal of Visualized Experiments*.

[37] Hoenicke L., Zender L. (2012). Immune surveillance of senescent cells--biological significance in cancer- and non-cancer pathologies. *Carcinogenesis*.

[38] Tchkonia T., Zhu Y., Van Deursen J., Campisi J., Kirkland J. L. (2013). Cellular senescence and the senescent secretory phenotype: therapeutic opportunities. *Journal of Clinical Investigation*.

[39] Freund A., Orjalo A. V., Desprez P.-Y., Campisi J. (2010). Inflammatory networks during cellular senescence: causes and consequences. *Trends in Molecular Medicine*.

[40] Muñoz-Espín D., Serrano M. (2014). Cellular senescence: from physiology to pathology. *Nature Reviews Molecular Cell Biology*.

